# The Exercise Treatment of Locomotor Ataxia

**Published:** 1898-07

**Authors:** 


### THE EXERCISE TREATMENT OF LOCOMOTOR ATAXIA.

The treatment of locomotor ataxia by exercises calculated to teach the patient again the co-ordination of muscles that has been lost by degeneration of the lower sensory neurons has recently attracted considerable attention, and has won for itself the support of many noted neurologists, among whom may be mentioned Leyden, Jolly, Mendel, Eulenberg, Oppenheim, Gerhardt, and Remak. This method of treatment was first introduced by Fraenkel, and has for its prime object the conversion of the simplest ataxic movement into a normal one. In a communication to the *Deutsche medicinische Wochenschrift* for December 17, 1897, Fraenkel describes the various exercises for the hands, arms, body and legs. For exercising the upper extremities the following directions are given: Sit in front of a table, place the hand upon it, then elevate each finger as far as possible; raising the hand slightly, extend, and then flex each finger and thumb as far as possible; do this with the right hand and then with the left hand. Touch with the end of the thumb each finger-tip separately and accurately; then touch the middle of each phalanx with the tip of the thumb. Sit at the table with a large sheet of paper and a pencil; make a dot at each corner of the paper and one in the center, and draw lines from the corner dots to the center dot, first with the right and then with the left hand. Put ten coins on the paper, pick them up and place them in a single pile, first with the right and then with the left hand. For the body and legs sample exercises: Sit in a chair, rise slowly to erect position without help of cane or arms of chair; then sit down slowly; stand with cane, feet together; advance left foot and return it; then the same with right. Walk slowly ten steps forward and five back with help of cane. Stand without cane, but with the feet a little apart and the hands on the hips; in this position stoop down by flexing the knees, and rise slowly. Stand without cane with the feet separated; raise the hands from sides above the head; carry them downward and foward, and try to touch the toes. Walk along a fixed line on the floor by help of a cane, placing each foot in turn on the line; then repeat without using the cane. Most of these exercises should be repeated several times, and the movements should be made with the eyes both open and closed.

Owing to disturbance of the sensory paths tabetics have lost the sense of fatigue, so there is some danger in overdoing the treatment. Two things are therefore insisted upon: first, every movement must be done with the greatest possible exactitude, since it is not simply physical exercise that is aimed at so much as training in co-ordination; and, second, the *seance* should not last more than eight or ten minutes, and no more than two should be allowed a day.

In the preataxic stage the exercise treatment has in a number of reported cases prevented the development of inco-ordination. Even in advanced sclerosis remarkable results may be obtained; in a number of instances patients, bedridden for three, four, and five years, have been taught to walk without assistance. The improvement may last for years, if the disease is stationary or only slowly progressive. According to Fraenkel the treatment is absolutely contraindicated in cases of acute or subacute ataxia.

Kalinin (*Vratch*, No. 7, 1897), who has used Fraenkel's method in five cases of locomotor ataxia, draws the following conclusions: By this treatment the loss of motion can be restored to a satisfactory degree, the gait and locomotion gradually becoming safer and firmer. The sense of locality and that of movement, and the skin sensibility, are but little improved. Romberg's symptom very soon became less pronounced. The duration of treatment should entirely depend upon the prognosis and the degree of the motor disturbances present, but in any case it should not be less than a month. No ill effects were observed when the treatment was interrupted at short intervals of two or three weeks, but not longer.

Raichline, who has treated twelve cases with complete success in eight, concludes that the conditions of success are a long, as opposed to a short, course of treatment, a well-nourished condition, good sight necessary for watching the movements accurately, a certain amount of energy and intelligence, not complete loss of sensibility, and the absence of arthropathies.—*University Medical Magazine.*

